# Early Radiological Limitations in Bone Healing Estimation After Allogeneic Bone Grafts Used for Mandible Reconstruction

**DOI:** 10.3390/diagnostics15212724

**Published:** 2025-10-27

**Authors:** Kamil Nelke, Klaudiusz Łuczak, Maciej Janeczek, Mikołaj Włodarczyk, Magdalena Florek, Małgorzata Tarnowska, Agata Małyszek, Cyprian Olchowy, Maciej Dobrzyński, Piotr Kuropka

**Affiliations:** 1 Facial Surgery Ward, EMC Hospital, Pilczycka 144, 54-144 Wrocław, Poland; 2 Department of Biostructure and Animal Physiology, Wrocław University of Environmental and Life Sciences, Cypriana K. Norwida 31, 50-375 Wrocław, Poland; maciej.janeczek@upwr.edu.pl (M.J.);; 3 Division of Microbiology, Department of Pathology, Wrocław University of Environmental and Life Sciences, Cypriana K. Norwida 25, 50-375 Wrocław, Poland; 4 Collegium Medicum, Jan Dlugosz University in Czestochowa, Armii Krajowej 13/15, 42-217 Czestochowa, Poland; 5 Department of Pediatric Dentistry and Preclinical Dentistry, Wrocław Medical University, Krakowska 26, 50-425 Wrocław, Poland; maciej.dobrzynski@umw.edu.pl

**Keywords:** odontogenic cyst, allogeneic bone, allograft, mandible, reconstruction

## Abstract

Xenograft bone, autologous bone grafts or allogeneic bones from a bone bank are used for bone augmentation, reconstruction or other purposes, when the volume, shape, and size of each jawbone defect require different bone materials. In the case of some bigger and locally advanced bone defects, the use of allogeneic bone can be suitable and used with great success if the wound and bone are especially carefully maintained; however, the healing period of each bone depends on good and stable wound closure followed by improved local antiseptic protocol. The individuality of each bone defect might also require additional prophylactic titanium plating in order to decrease the risk of possible mandibular fracture or to help improve bone stability, reduce bone mobility and possible inflammation or granulation tissue formation. Early radiological estimation of bone healing evaluation might be troublesome and not fully visible in radiological evaluation in the early stages of bone healing. On the other hand, possible bone inflammation, radiolucent defects, and granulation formation could be noted in cases of acute or long-lasting bone grafting material inflammation, bacterial contamination within the bone defect area, or the presence of fistula. The presented case describes a very good outcome from a dentigerous cyst removal with bone defect grafting and plating; however, because of wound dehiscence and allogeneic bone graft exposure, the patient required one additional procedure.

**Figure 1:**
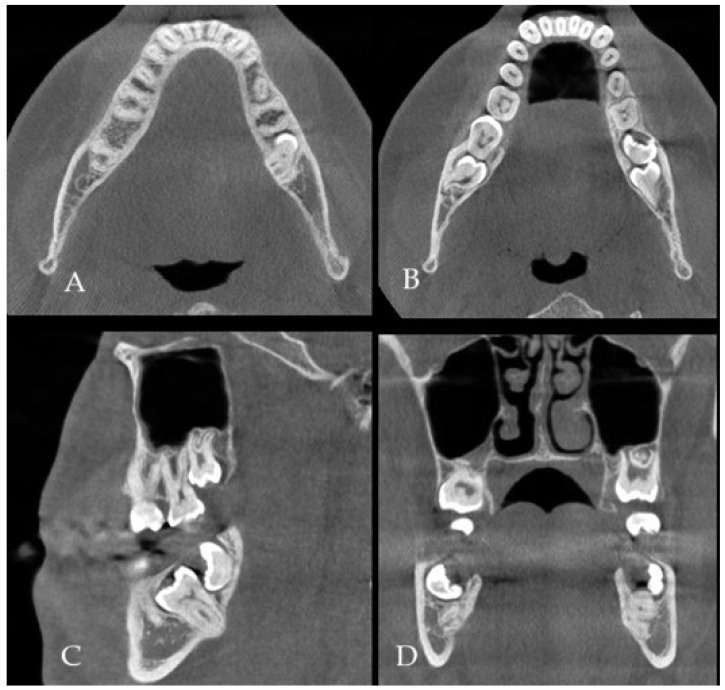
(**A**) CBCT (cone beam computed tomography scans: CBCT 20 × 20 cm FOV (field of view) imaging protocols based on RayScan S 5471.3 mGy (Ray Company Co., Ltd., Samsung 1-ro, Hwaseong-si, Gyeonggi-do, Republic of Korea), CBCT evaluation was carried out in RAYSCAN S without slicing, with an average thickness between 0.070 mm and 0.3 mm) is a very valuable diagnostic tool to estimate bone status, shape, and its anatomy, as well as the boundaries of the dentigerous cyst (DC) and impacted or retained teeth. The authors used a suitable software for CBCT evaluation (RadiAnt Dicom Viewer Software version 2020.2.1 Medixant, Poznań, Poland). In some cases, after the removal of any teeth and cysts or tumors within the jaw bones, it is quite common that due to the bone loss, a fracture of the mandible or some degree of bone mobility might be found. Some authors in those cases suggest not only a prophylactic bone plating with titanium miniplates but also applying some additional bone grafts to improve bone volume and avoid any early or late bone fracture. Both a bone fracture and its mobility after the removal of a certain portion of bone might not only cause its delayed healing, resulting in a complete or displaced fracture but also promote some granulation tissue, local inflammation, or fistula occurrence. A good CBCT evaluation is helpful in the planning of the scope of each surgery and in predicting the occurrence of some worrisome aspects, especially related to bone loss, fracture occurrence, and planning for any orthodontic treatment. The type of bone used (xenograft, allograft, autograft) could be used in various bone defects. In the presented case, because the occurrence of totally impacted teeth 37 and 38 with a presence of DC, a decision was made to use an allograft bone ((**A**–**D**), where (**A**,**B**) axial scans, (**C**)—sagittal view and (**D**)—coronal view in CBCT). Because of the level of teeth 37 retention, its proximity to the lower mandible border, and perforation of both lingual and buccal plates, a larger amount of bone was necessary to stabilize the mandible left basis deficit and improve its postoperative height and width. On the other hand, a so-called PMP (prophylactic/preventive mandible plating) during the removal of more locally advanced jaw lesions is a good solution. A panoramic radiograph (panx) or a classic CT does not show the close boundaries between tooth roots, inferior alveolar nerve (IAN), cortical plate, and the scope of DC occurrence, but the panx role for screening is quite good [1,2,3].

**Figure 2:**
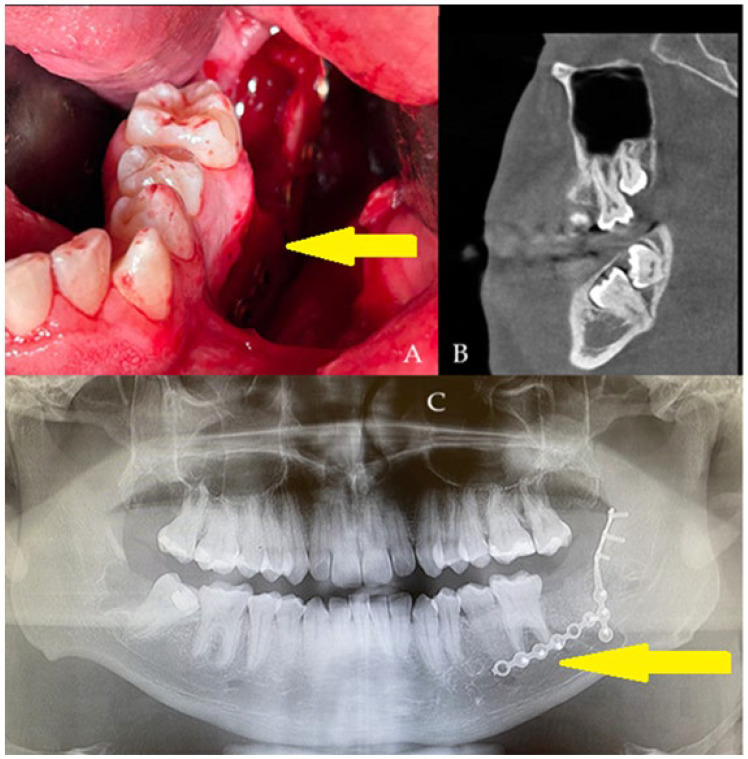
A generally healthy 20-year-old male was scheduled for surgery under general anesthesia and nasotracheal intubation. The oral cavity was free of the intubation tube, which was sutured to the nares, just in case of any mandibular fracture, the necessity of setting proper occlusion or applying additional devices, if they would be necessary. All wisdom teeth 18, 28, 48, with impacted 37, 38 ((**B**), sagittal view) were removed for orthodontic purposes and scheduled treatment of malocclusion. Typical surgical tooth removal was uneventful; however, because of slight bending of both cortical plates, additional titanium plates were used (yellow arrow, (**C**)) (2.0 System, Medartis, Basel, Switzerland). To improve PMP outcomes, an additional 10 cm^3^ allogeneic bone graft (FFABG, fresh–frozen allogeneic bone graft, *RCKiK*, Katowice, Poland) was placed in the bone deficit to improve the healing period, and then a layer-by-layer suture technique for both lingual and buccal flaps was used to cover the deficit tightly (**A**). In the first seven days, a postoperative panx revealed a very good titanium plate position, along with a stable bone graft position not exceeding the alveolar ridge. After two weeks, a slight wound dehiscence just behind tooth 36 was noted. Local treatment of the wound and its debridement was not scheduled because the patient denied any revision or local surgery protocol. The wound decreased in size over time. The patient was clear of inflammation and pus formation, and no more bone was visible; a slight wound was left for secondary healing. The allogeneic bone graft requires very careful usage, improved oral hygiene, and very gentle wound care [4,5].

**Figure 3:**
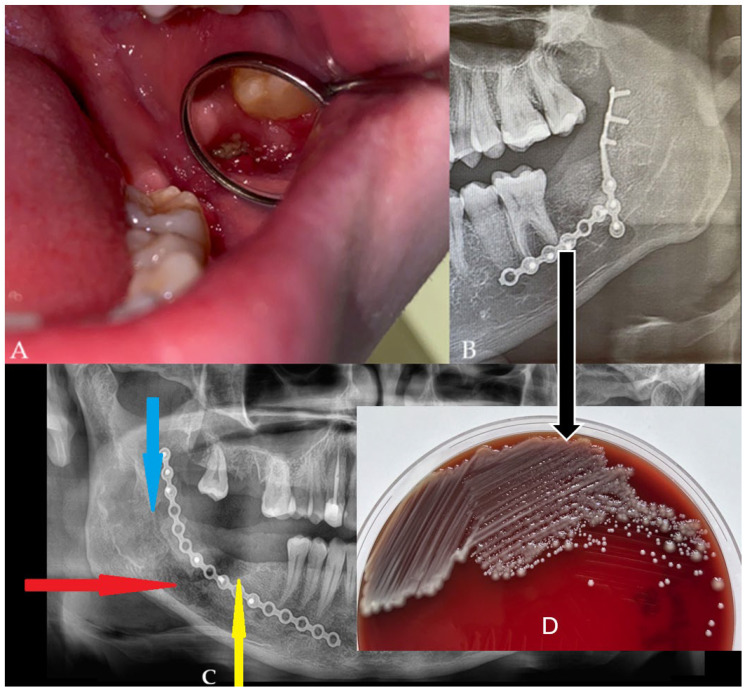
Six months after surgery, the wound still had a small dehiscence (**A**). No fluid accumulation was seen; however, the patient reported cases of some loose bone secretion. Control panx revealed good bone volume, proper healing in the deeper layers, no formation of granulation tissue, nor any bone inflammation or signs of bone resorption or any radiolucent area in the grafted bone (**B**). A different comparable case of a 51-year-old male with hypertension and with active inflammation is presented in (**C**). In the main case (**B**), the used plating systems were stable without any local bone changes. The used panx is very limited in establishing full bone changes; however, it is suitable enough for early screening purposes for bone changes estimation. In the presented case, without any presence of bone loss, bone resorption, formation of cystic cavities, bone inflammation, or the presence of granulation tissue with partially unhealed or necrotic bone parts and without typical halo marks and radiolucencies, there was no necessity for additional CBCT evaluation (**A**,**B**). The patient (**B**) was asymptomatic, and the wound had been stable for the past six months. The patient finally decided on a revision surgery and wound debridement. Before the surgery, a microscopic specimen evaluation from the wound revealed colonization of part of the bone with *Klebsiella pneumoniae* and *Staphylococcus aureus* (**D**). Intraoral Biseptol (2 × 980 mg per day, sulfamethoxazole with trimethoprim, Adamed Pharma S.A., Czosnów, Poland) was used for 10 days, followed by local antiseptic agents three times per day, 0.1% CHX solution (gluconate chlorhexidine, Eludril, Pierre-Fabre Oral Care, Paris, France), and 3% H_2_0_2_ (hydrogen peroxide, Farmina, Kraków, Poland) as preparation for revision surgery. The lack of any worrisome radiological signs in a typical panx is a very good finding, especially the appearance of bone healing was very good without any worrisome factors, bone loss, and lack of its remodeling, suggesting an uneventful bone grafting procedure. In a six-month time frame, a very good remodeling process was established regardless of the constant presence of a dehiscence and unhealing wound in the oral cavity. This case presents how an oral wound (**A**) does not correlate entirely with any radiological changes in a six-month time frame (**B**). Furthermore, any small local soft and bone tissue problems do not seem to correlate with other changes, which seems to correspond to the fact that just major and more advanced bone inflammation (red arrow, comparable case—(**C**)), necrosis or local loss of healing, scattered radiolucencies (blue arrow) with bone loss are visible in the radiological evaluation, especially when a bigger scope of bone is changed and inflamed (yellow arrow), as presented in the comparable case (**C**). The loss of proper bone structure in the early six months of panx visualization is a very troublesome factor, which requires both improved diagnostics with CBCT and revision surgery with microscopic and histopathologic specimen evaluation. When a purulent or granulation fistula is present, serious bone inflammation with loss of bone volume is mostly present. Regardless of the type of panx used and CBCT, without any progression of worrisome symptoms, a typical screening with a panx radiograph is sufficient. Both presented cases required vision surgery with microbiological and histopathological wound evaluation. Patient (**B**) was scheduled for a revision surgery that consisted of wound debridement, curettage, and ostectomy until the levels of healed and bleeding bone were noted. Some samples were taken for both microbiological and histopathological evaluation. When wound healing is not sufficient in each case, both improved radiology with CBCT or histopathology should be taken to fully evaluate the scope of the problem in the wound, to evaluate whether it is related to the surgical procedure, bone inflammation, microbiological contamination, or if there is the necessity for a wound debridement or bone removal.

**Figure 4:**
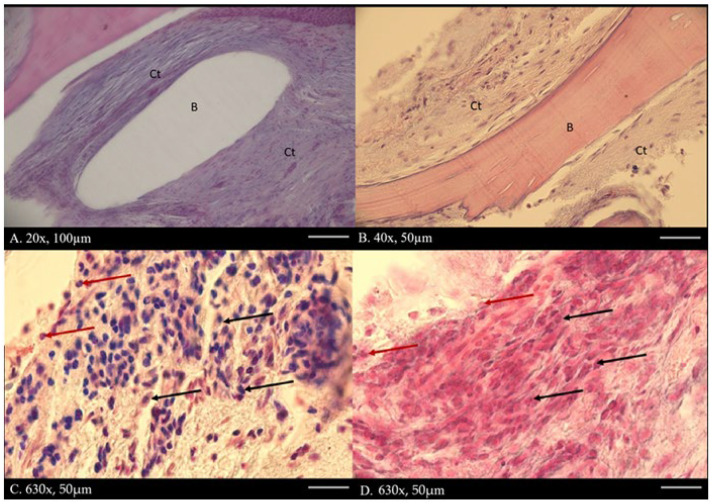
Main histological findings include fibrous tissue encasing portions of the graft, indicating an attempt by the host to isolate the bone. No multinucleated giant cells are present around residual graft material, reflecting a foreign body response, indicating no bone resorption. No extensive areas of necrotic bone are visible; however, decellularized bone is characterized by empty lacunae and loss of cellular detail. Surrounding soft tissue shows a solid structure surrounding bone fragments. Dense infiltration of neutrophils in the surrounding connective tissue dominates the graft site, indicating acute bacterial infection. Mixed populations of macrophages, lymphocytes, and plasma cells are also present, suggesting a chronic inflammatory component. The capsule surrounding the bone graft (**A**,**B**), where there is B-bone, Ct-connective tissue, and H-E staining. No necrotic zones adjacent to graft surfaces may be spotted. Capillary proliferation and endothelial swelling are not noted, but overall vascularization is poor, contributing to graft rejection and infection persistence. The presence of inflammation lymphocytes and macrophages (**C**,**D**) (black arrow) and neutrophils (red arrow) was found in connective tissue under 630× magnification with H-E staining.

**Figure 5:**
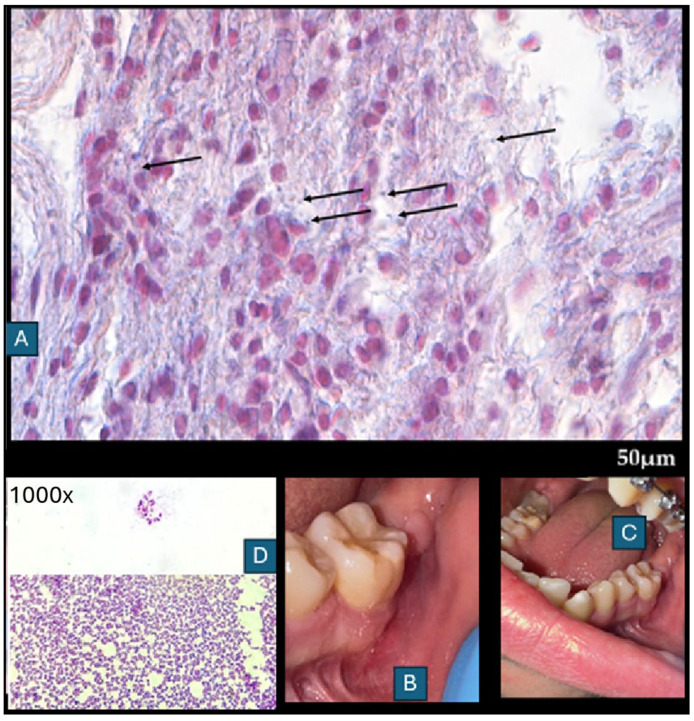
Postoperative histopathology (**A**) was compared with microbiological evaluation (**B**). The following paper illustrates a very clear radiographic–histologic mismatch in the selected case, because radiographic stability does not guarantee microbiological or histopathological healing. In connective tissue under 630× magnification with H-E staining, with differentiation for Gram-positive/-negative bacteria (1000×). Black arrows show Gram-positive bacteria. The clinical specimen obtained was subjected to microscopic examination using Gram staining. Then, it was inoculated onto blood agar (Columbia agar base agar, Oxoid Ltd., Basingstoke, UK; supplemented with 5% sheep blood), MacConkey agar (Oxoid), and Sabouraud dextrose agar (SDA, Oxoid), and incubated for 48 h at 37 °C (30 °C for SDA), under aerobic and anaerobic conditions. Grown cultures were analyzed phenotypically (colony morphology, Gram reaction, catalase activity), and chosen single colonies were subcultured under the same conditions. After obtaining pure cultures, DNA was isolated (Genomic Mini, A&A Biotechnology, Gdańsk, Poland), and the bacteria were identified by sequencing a fragment of the 16S rRNA gene, using PCR primers 16S-27f and 16S-907r. Gram-positive cocci in pairs or forming chains were observed in the preparations ((**D**)—direct specimen staining (top) and made of culture of *S. anginosus* (bottom)) from the microbiological scrub taken during the final surgery. Moderately abundant and mixed bacterial growth was detected. Three types of colonies were present in both aerobic and anaerobic cultures. Among the isolates tested via 16S rRNA gene sequencing, the two most abundant (one from aerobic and one from anaerobic culture) were recognized using the Basic Local Alignment Search Tool (https://blast.ncbi.nlm.nih.gov/Blast.cgi, accessed on 1 September 2025) as *Streptococcus anginosus* (100% identity with sequences listed in the GenBank database). Another three were assigned to the species *Streptococcus mitis* (identity 99.05–100%). The sequence of the remaining one was paired with one belonging to an uncultured bacterium, and the next best match was *Streptococcus oralis* (98.02%). All the isolated bacteria are regarded as commensals inhabiting, among others, the oral cavity. These microorganisms; however, under certain conditions, are able to cause opportunistic infections. Additional histochemical test revealed the presence of Gram-positive bacteria, which were inducing a mild immune response, associated with activation of the macrophage–lymphocyte system, as well as the presence of neutrophils. Inflammation appears to be essential for wound healing, with no evidence of bone resorption, granulation tissue, or inflammatory bone infiltration. Gram staining revealed no bacteria in the bones; they were present only in the adjacent tissues. The bacterial infection was not associated with the allograft. In relation to the above, no changes were observed in the radiological image of the bone (Figure 3B,C). Wound healing after debridement was uneventful (**B**,**C**). Because of the changes in microbiological scrubs from the wound, the patient continued intraoral antibiotics consisting of Amoxiclav Quick Tab (Amoxicillinum, Acidum clavulanicum, 2 × 1 g—Sandoz Poland, Warsaw, Poland) after the surgery for seven days. The following case presents how bone healing in early stages up to six months does not correspond with clinical, radiological, and microbiological findings. This might be found because of a very local and small wound without any progression and invasion of deep layers of grafted FFABG bone in both clinical and radiological examination. The disproportion between clinical and radiological appearance when comparing the healing is not equal to the bone healing in gathered histopathological samples, nor is it free of bacterial colonies, regardless of the pharmacological agents used. Histopathological examination revealed parts of healed bone surrounded by unhealed necrotic bone, surrounded by cyst-like thin-layer pathologies with bacteria scattered along the different layers of the sample. It seems that the role of saliva, oral biofilm, and adjacent local oral factors has a great effect on the bone. Secondly, and possibly, bacterial influence on the FFABG is higher when the bone is exposed to the oral cavity and not covered by a flap. The mixture of neutrophile, macrophage, and lymphocytic specimens suggests an unspecified inflammation with some swelling and cyst-like mass when the deeper layer was fully healed (hard bleeding bone with full remodeling evaluated during revision surgery). The presented process suggests that the wound and the patient’s body tried to get rid of and remove the entire unhealed bone on their own and decrease any potential unwanted results on their own. The presence of a connective tissue capsule surrounding a radiologically healthy bone was not seen in the panx radiograph. In radiological images, the bone was healing properly, but in microscopic evaluation, the superficial layer of bone was not healing and was not fully remodeling in the mentioned six-month time frame. Radiological evaluation did not reveal any granulation tissue, empty cystic-like cavities, bone resorption, or any worrisome radiological symptoms. Radiology alone is not sufficient to establish bone healing, bone remodeling, and possible bone infection with bacteria in its early stages, when the patient’s local or general symptoms do not reflect any active inflammation, granulation tissue, or pus formation in clinical examination [6,7]. Any early inflammation processes in the used FFABG are difficult to establish in any early radiological detection. Based on the histopathological evaluation, any late stages can be more successfully identified in routine panx, as presented in a comparable case (Figure 3C). Local bone swelling with inflammatory reaction is normally visible more accurately in early radiographs. Perhaps due to the extensive usage of local agents and antibiotics for specific microflora, the scope of infection and bone changes was limited to a very small portion of bone that could not be evaluated and established in such early stages of healing. When some troublesome or worrisome radiological (panx) or clinical symptoms arise, improved diagnostics with CBCT should assess the bone healing and remodeling more accurately if there is a necessity for such a step. Secondly, wound dehiscence might happen in each stage of healing and the surgery period, but as it seems in this case, local wound care and local appliances might have improved the final outcome [8,9,10]. Based on the following cases, the authors recommend that (1) each allogeneic bone should be closely monitored; (2) a potential need for combined imaging and microbiological surveillance should be considered in wound dehiscence and lack or prolonged healing; (3) any sole reliance on panoramic radiography alone should be limited; (4) each individual patient case improved oral hygiene protocol before and after surgery helps in decreasing possible worrisome healing; (5) microbiological scrubs help a lot in wound treatment in the oral cavity.

## Data Availability

The datasets used and/or analyzed during the current study are available from the corresponding author upon reasonable request (histology images, microbiology raw data, additional CBCT, RTG scans).
